# Transcriptional and microbial profile of gastric cancer patients infected with Epstein-Barr virus

**DOI:** 10.3389/fonc.2025.1530430

**Published:** 2025-03-05

**Authors:** Klezzer de Oliveira Carneiro, Taíssa Maíra Thomaz Araújo, Ronald Matheus Da Silva Mourão, Samir Mansour Moraes Casseb, Samia Demachki, Fabiano Cordeiro Moreira, Ândrea Kely Campos Ribeiro Dos Santos, Geraldo Ishak, Daniel de Souza Avelar Da Costa, Leandro Magalhães, Amanda Ferreira Vidal, Rommel Mario Rodriguez Burbano, Paulo Pimentel de Assumpção

**Affiliations:** ^1^ Oncology Research Center, Federal University of Pará, Belém, Brazil; ^2^ Biological Sciences Institute, Federal University of Pará, Belém, Brazil; ^3^ Ophir Loyola, Federal University of Pará, Belém, Brazil

**Keywords:** gastric cancer, EBV, metatranscriptomics, gastric microbiome, carcinogenesis

## Abstract

**Introduction:**

Gastric cancer (GC), which has low survival rates and high mortality, is a major concern, particularly in Asia and South America, with over one million annual cases. Epstein-Barr virus (EBV) is recognized as a carcinogen that may trigger gastric carcinogenesis by infecting the stomach epithelium via reactivated B cells, with growing evidence linking it to GC. This study investigates the transcriptional and microbial profiles of EBV-infected versus EBV-non-infected GC patients.

**Methods:**

Using Illumina NextSeq, cDNA libraries were sequenced, and reads were aligned to the human genome and analyzed with DESeq2. Kegg and differential analyses revealed key genes and pathways. Gene sensitivity and specificity were assessed using ROC curves (p < 0.05, AUC > 0.8). Non-aligned reads were used for microbiome analysis with Kraken2 for bacterial identification. Microbial analysis included LDA score, Alpha and Beta diversity metrics, with significance set at p ≤ 0.05. Spearman’s correlation between differentially expressed genes (DEGs) and bacteria were also examined.

**Results:**

The data revealed a gene expression pattern in EBV-positive gastric cancer, highlighting immune response, inflammation, and cell proliferation genes (e.g., *GBP4*, *ICAM1*, *IL32*, *TNFSF10*). ROC analysis identified genes with high specificity and sensitivity for discriminating EBV+ gastric cancer, including *GBP5*, *CMKLR1*, *GM2A* and *CXCL11* that play pivotal roles in immune response, inflammation, and cancer. Functional enrichment pointed to cytokine-cytokine receptor interactions, antigen processing, and Th17 immune response, emphasizing the role of the tumor microenvironment, shaped by inflammation and immunomodulation, in EBV-associated GC. Microbial analysis revealed changes in the gastric microbiota in EBV+ samples, with a significant reduction in bacterial taxa. The genera *Choristoneura* and *Bartonella* were more abundant in EBV+ GC, while more abundant bacteria in EBV- GC included *Citrobacter*, *Acidithiobacillus* and *Biochmannia*. Spearman’s correlation showed a strong link between DE bacterial genera and DEGs involved in processes like cell differentiation, cytokine production, digestion, and cell death.

**Conclusion:**

These findings suggest a complex interaction between the host (EBV+ GC) and the microbiota, possibly influencing cancer progression, and offering potential therapeutic targets such as microbiota modulation or gene regulation. Comparing with EBV- samples further highlights the specific impact of EBV and the microbiota on gastric cancer pathogenesis.

## 1 Introduction

Gastric cancer (GC) is a lethal disease with low overall survival rates worldwide. The majority of new annual GC diagnoses primarily occur in Asian and South American countries (1) and remain a significant public health issue, with over 1 million new cases each year globally. According to data from the International Agency for Research on Cancer in 2022, gastric cancer ranked fifth among the most frequent cancers worldwide in both sexes, representing 4.8% (968,784 cases) (2). Furthermore, gastric adenocarcinoma accounts for over 90% of cases of this neoplasm (3). In 2018, GC incidence rates were nearly twice as high in men as in women (4).

In Brazil, GC was the sixth most frequent cancer (23,021 cases) in 2022 (5). It was estimated that, for each year of the 2020-2022 triennium, 13,360 new cases of stomach cancer occurred among men and 7,870 among women. These values correspond to an estimated risk of 12.81 per 100,000 men and 7.34 per 100,000 women (6).

In this context, the gastric microbiota has emerged as a relevant factor in carcinogenesis. The gastric microbiota, comprising a diverse community of microorganisms, plays a crucial role in stomach health and is closely linked to the development of gastric cancer. Studies suggest that dysbiosis of the gastric microbiota, particularly an increased presence of *Helicobacter pylori*, contributes to chronic inflammation and damage to the gastric epithelium, promoting conditions conducive to carcinogenesis (7).

Furthermore, the association of gastric cancer with viral infections, such as Epstein-Barr virus (EBV), indicates a significant role for microbial factors in the onset and progression of gastric cancer, with growing evidence linking EBV to gastric carcinogenesis (8). Understanding the gastric microbiota thus opens possibilities for novel therapeutic and preventive approaches (9).

The frequency and distribution of gastric cancer subtypes, as well as the prevalence of *H. pylori* and EBV, vary across different geographic regions worldwide. The rate of EBV infection in gastric cancer ranges between 4.3% and 18% (10).

An increasing number of reports suggest cooperation between EBV and *H. pylori*, where the presence of one microorganism may promote the growth or virulence of the other. Although the mechanisms governing this synergistic interaction are not fully understood, evidence suggests that coinfection with *H. pylori* and EBV significantly increases immune cell recruitment to the infection site, enhancing gastric inflammation and tissue damage. For instance, monochloramine, an oxidant produced in the stomach during *H. pylori* infection, may trigger the transition of EBV from a latent to a lytic phase (11).

Other pro-inflammatory cytokines emerging from *H. pylori*-induced gastric inflammation may facilitate EBV proliferation. For example, *H. pylori*-induced secretion of interferon γ (IFN-γ) promotes an inflammatory environment that exacerbates disease severity (12). Interleukins 6 and 13 (IL-6 and IL-13) promote EBV proliferation, and elevated levels of pro-inflammatory cytokines such as IL-1β, tumor necrosis factor α (TNF-α), and IL-8 have been observed in EBV and H. pylori coinfection associated with severe gastritis (13). Consistent with these findings, plasma IFN-γ levels in gastric cancer patients correlate positively with the extent of EBV reactivation (14).

Additionally, persistent activation of Th17 cells appears to contribute to gastric inflammation associated with *H. pylori* and EBV coinfection. Th17 cells, a subset of pro-inflammatory CD4+ T helper cells, activate innate immune cells, regulate B cell responses, and are involved in antimicrobial immune responses and wound healing. Th17 cells and their key cytokine, IL-17A, are implicated in the pathogenesis of *H. pylori-*induced gastritis (15).

Regarding the resident gastric microbiota, recent studies have detected microbiome alterations in gastric cancer compared to non-cancer individuals, indicating dysbiosis, with distinct characteristics that can differentiate GC from other diseases (16). Bacteria found in gastric cardia adenocarcinoma tissues primarily belong to the Firmicutes, Bacteroidetes, and Proteobacteria phyla (17).

At the bacterial genus level, significant increases in the abundance of *Achromobacter*, *Citrobacter*, *Phyllobacterium*, *Clostridium*, *Rhodococcus*, and *Lactobacillus* have been observed in GC compared to chronic gastritis (18). Additionally, a marked difference in microbiota composition exists between non-atrophic gastritis and GC, with bacterial diversity gradually decreasing from non-atrophic gastritis to intestinal metaplasia, and finally to cancer. Notably, *H. pylori*, *Bacteroidetes* and *Fusobacteria* abundance decreases while commensal intestinal bacteria increase (18, 19).

In this context, the present study aimed to investigate the transcriptional and microbial profiles of EBV-infected GC patients, compared to EBV-non-infected GC patients, in order to describe the profile of human transcripts in GC EBV infection, correlate differentially expressed GC genes with EBV positivity and characterize microbiome of EBV+ GC.

## 2 Methods

### 2.1 Patients, sample characterization and ethical considerations

Forty-one samples of stomach tumor tissue were collected from patients diagnosed with gastric adenocarcinoma, of which 8 are from patients with GC positive for EBV (GC EBV+) and the remainder were negative for the virus (GC EBV-). None of the EBV-positive samples show co-infection with *H. pylori*.

The patients included in this study had their medical records and clinical data reviewed for the presence of gastric adenocarcinoma, sex, age, Laurén histological subtype, and *H. pylori* and EBV infections (**Table 1**). The individuals were recruited from João de Barros Barreto University Hospital (HUJBB) at the Federal University of Pará (UFPA).

**Table 1:** Clinicopathologial data of patients.

Samples	Age	Gender	TNM	Laurén	Location	EBV	*H. pylori*
1	82	M	II	Intestinal	Noncardia	Negative	Negative
2	65	M	III	Intestinal	Noncardia	Negative	Negative
3	51	M	II	Intestinal	Noncardia	Negative	Negative
4	65	M	II	Intestinal	Noncardia	Negative	Negative
5	62	M	IV	Intestinal	Cardia	Negative	Negative
6	63	F	III	Diffuse	Noncardia	Negative	Negative
7	66	F	II	Intestinal	Noncardia	Negative	Negative
8	58	M	IV	Intestinal	Noncardia	Negative	Positive
9	56	M	IV	Intestinal	Noncardia	Negative	Negative
10	59	M	IV	Intestinal	Noncardia	Negative	Negative
11	64	M	II	Intestinal	Noncardia	Negative	Negative
12	55	F	II	Intestinal	Noncardia	Negative	Negative
13	66	F	II	Intestinal	Noncardia	Negative	Positive
14	54	M	III	Intestinal	Noncardia	Negative	Negative
15	53	M	IV	Intestinal	Noncardia	Negative	Negative
16	59	M	IV	Intestinal	Noncardia	Negative	Negative
17	70	M	II	Intestinal	Noncardia	Negative	Positive
18	72	F	III	Diffuse	Noncardia	Negative	Negative
19	73	M	III	Intestinal	Noncardia	Negative	Negative
20	72	M	III	Diffuse	Noncardia	Negative	Positive
21	48	M	I	Intestinal	Noncardia	Negative	Positive
22	55	M	III	Intestinal	Noncardia	Negative	Positive
23	46	F	III	Diffuse	Noncardia	Negative	Negative
24	67	M	IV	Intestinal	Noncardia	Negative	Negative
25	34	F	I	Diffuse	Noncardia	Negative	Positive
26	48	F	II	Diffuse	Noncardia	Negative	Positive
27	38	F	III	Diffuse	Noncardia	Negative	Negative
28	64	M	III	Diffuse	Noncardia	Negative	Negative
29	70	M	III	Intestinal	Cardia	Negative	Negative
30	49	F	II	Diffuse	Noncardia	Negative	Positive
31	61	F	I	Diffuse	Noncardia	Negative	Positive
32	74	F	III	Diffuse	Noncardia	Negative	Positive
33	70	F	I	Intestinal	Noncardia	Negative	Negative
34	58	M	IV	Intestinal	Noncardia	Positive	Negative
35	55	M	II	Intestinal	Noncardia	Positive	Negative
36	38	F	IV	Diffuse	Noncardia	Positive	Negative
37	37	M	II	Intestinal	Noncardia	Positive	Negative
38	54	F	III	Intestinal	Cardia	Positive	Negative
39	74	F	III	Intestinal	Noncardia	Positive	Negative
40	52	M	III	Intestinal	Noncardia	Positive	Negative
41	63	M	III	Intestinal	Noncardia	Positive	Negative

All participants were thoroughly informed about the study’s objectives and consulted regarding their participation. Samples were only collected after obtaining informed consent through the Free and Informed Consent Form (TCLE). The use of all samples and the conduct of this study were approved by the Ethics Committee for Research of the João de Barros Barreto University Hospital, under CAAE number 47580121.9.0000.5634.

During tumor sample collection, a 0.5 cm fragment of tissue was excised. These fragments were immediately collected post-gastric resection, stored in RNA later for transport, and subsequently preserved at –80°C.

### 2.2 Total RNA extraction and library preparation for sequencing

Initially, approximately 50–100 ng of tissue from each sample were homogenized. Following this, 1 ml of TRIZOL^®^ reagent (Thermo Fisher Scientific) was added to the processed tissue for RNA extraction. TRIZOL^®^ was used to maintain RNA integrity and facilitate cell lysis. After centrifugation at 13,000 rpm for 10 minutes at 4°C, RNA was precipitated with isopropyl alcohol. The resulting total RNA was washed with ethanol, air-dried, and kept at room temperature.

RNA integrity and concentration were analyzed using the Qubit 2.0 Fluorometer (Thermo Fisher Scientific), NanoDrop ND-1000 (Thermo Fisher Scientific), and the 2200 TapeStation System (Agilent). Ideal RNA integrity criteria were values between 1.8 and 2.2 (A260/A280 ratio), >1.8 (A260/A230 ratio), and RNA Integrity Number (RIN) ≥ 5. The total RNA obtained was stored at –80°C until further use.

For library construction, the TruSeq Stranded Total RNA Library Prep Kit with Ribo-Zero Gold (Illumina) was used according to the manufacturer’s instructions. A total of 1 μg of total RNA per sample in a 10 μL volume was used for library preparation. After construction, the libraries’ integrity was assessed again using the 2200 TapeStation System, revealing a final product band of ~260 base pairs.

### 2.3 RNA-seq and computational biology

The cDNA libraries were loaded onto the Illumina NextSeq sequencing system and sequenced using paired-end reads. The NextSeq^®^ 500 ID Output V2 Kit - 150 cycles (Illumina) was employed according to the manufacturer’s guidelines.

Base-calling was performed, and the reads were converted to FASTQ format using the Reporter software, encoding sequence and quality scores in ASCII. Read quality was then visualized in FastQC v0.11.9. Low-quality reads and adapters were removed using Trimmomatic (20).

Filtered reads were aligned to the human transcriptome using hg v38 as a reference index for coding transcripts (18, 19, 21) and quantified using Salmon v1.5.2 (22). Reads were imported from Salmon into R v4.1.0 (R: The R Project for Statistical Computing) with the Tximport v3.14.0 package (23). DESeq2 v3.14 (24) was used to estimate gene-level abundances based on Tximport data. Differentially expressed gene threshold was set to |log2FoldChange| ≥ 1 and adjusted p-value ≤ 0.05. Normalized gene levels was used in subsequent analyses.

To investigate potential pathways and biological functions associated with genes, functional enrichment analysis was conducted. KEGG was used through the ClusterProfiler v4.3.2 package in R (25), with enrichment terms considered significant at an adjusted p-value ≤ 0.05.

Receiver operating characteristic (ROC) curves were plotted for each gene’s expression using the pROC v1.18 package (26), with the area under the curve (AUC) calculated to assess each gene’s sensitivity and specificity for the clinically relevant differential expression.

Reads that were not aligned with the human genome were used for microbiome analysis and Kraken2 tool was used to perform bacterial identification. The differential analysis was carried out using the DESeq2 package from the R software, and the bacterial genus was considered differentially abundant (DA) when |log2FC| > 1 and adjusted p-value ≤ 0.05.

To assess the separation between the EBV-positive and EBV-negative groups based on bacterial abundances, the LDA score was computed using Linear Discriminant Analysis (LDA) from the MASS library on R. Statistical analyses between EBV-positive and EBV-negative LDA scores were performed using the Wilcoxon-Mann-Whitney test.

For the analysis of alpha diversity, Simpson and Shannon indices were used and for beta diversity, PERMANOVA, PCoA and the Bray-Curtis plot were used. All analyzes were carried out using Library Vegan in R, being considered significant when the p value ≤ 0.05.

Finally, Spearman correlation was performed between DE genes and DE bacteria. DE genes correlated (ρ > |0.3|) with at least 10% of DE bacteria were used for the correlation plot and gene ontology analysis. Gene Ontology was performed through the ClusterProfiler v4.3.2 package in R (25), with enrichment terms considered significant at an adjusted p-value ≤ 0.05.

## 3 Results

Approximately 6 million reads were obtained per sample, with an average of 18,000 human genes identified. Of these, around 5,000 reads were from bacteria, with an average of 600 bacterial genera per sample (**Supplementary Tables 1**, **2**).

No statistically significant correlations were found between clinicopathological data of patients and EBV status. Additionally, sample size did not allow pairing cases by location, besides there are cases with tumors occupying more than one region, limiting this type of analysis.

### 3.1 Differential expression analysis of human genes

**Figure 1:**
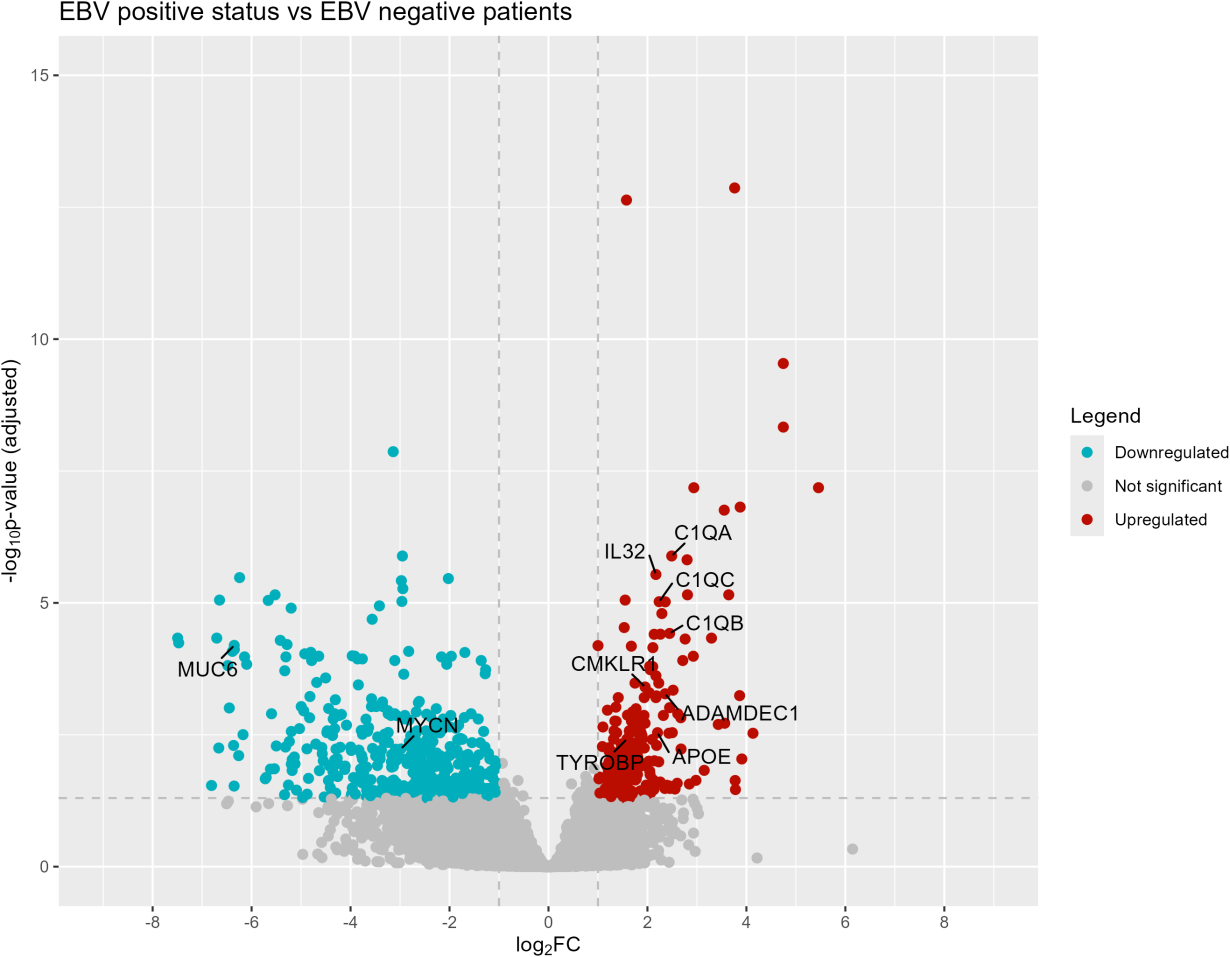
Volcano plot of differentially expressed genes in CG EBV+ versus CG EBV- samples.

The differential expression analysis, carried out by DESeq2 package in R, between EBV-positive GC and EBV-negative GC is presented in **Figure 1** (117 upregulated and 202 downregulated, **Supplementary Table 3**), where majority of human genes are found to be downregulated (light blue on the left), including the *MUC6* gene, associated with gastric mucus production, and *MYCN*, a transcription factor implicated in cell cycle control and proliferation.

Conversely, on the right side of the volcano plot, there is an overexpression of genes *C1QA, C1QB, C1QC, IL32, ADAMDEC1, CMKLR1, TYROBP*, and *APOE* in EBV-positive gastric cancer samples compared to EBV-negative samples (**Figure 1**), suggesting an enhanced activation of immune and inflammatory pathways, since *C1QA*, *C1QB* and *C1QC* are genes that encode three polypeptide chains that make up the complement C1q subcomponent and are positively correlated with immune cell markers, such as B cells, CD8+ T cells, CD4+ T cells, M1 macrophages and M2 macrophages, and *IL32* encodes a proinflammatory cytokine that is involved in the development of inflammatory diseases and malignancies.

**Figure 2:**
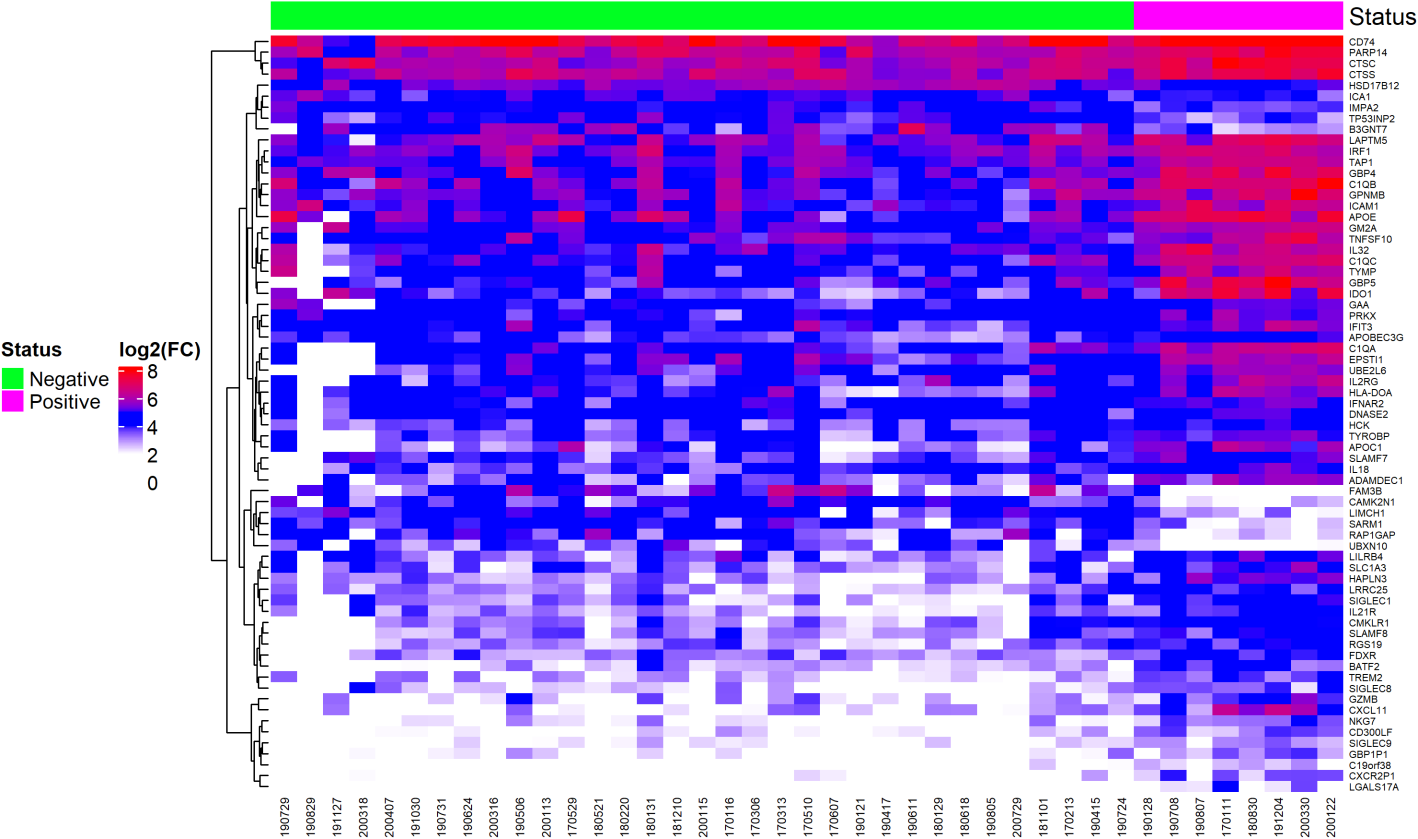
Heatmap of differentially expressed genes, selected by ROC curve analysis, showing EBV-positive and EBV-negative statuses.

The heatmap shown in **Figure 2** illustrates a cluster (highlighted in red) of differentially expressed genes in EBV+ GC samples. Key genes in this cluster include *LAPTM5, IRF1, TAP1, GBP4, C1QB, GPNMB, ICAM1, APOE, GM2A, TNFSF10, C1QC, TYMP, GBP5*, and *IDO1*. The white cluster highlights downregulated genes in EBV+ GC samples, with notable genes including *FAM3B, CAMK2N1, LIMCH1, SARM1, RAP1GAP*, and *UBXN10*.

Most of these genes are not discussed in literature and their role in EBV infection and cancer is not clear, except for *IRF1* and *FAM3B*, that function as tumor suppressor gene and oncogene, respectively. *IRF1* encodes a transcription factor stimulates an immune response against tumor cells and *FAM3B* encodes a protein called FAM3B/PANDER, which is a hormone that regulates glucose and lipid metabolism and its expression is associated with the progression of multiple types of cancer.

### 3.2 Receiver operating characteristic analysis

**Figure 3:**
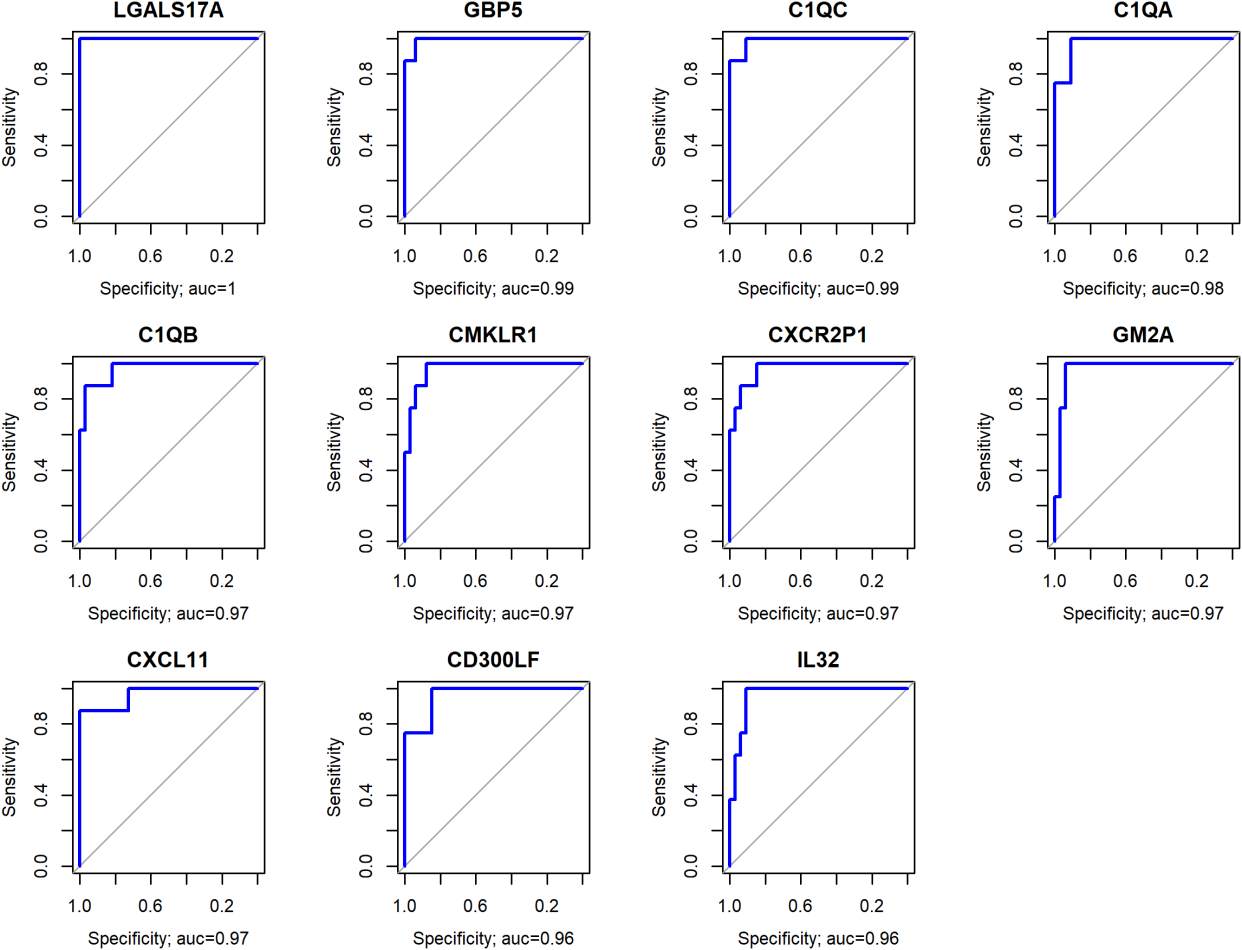
Genes with the highest AUC values, representing the optimal trade-off between sensitivity and specificity in distinguishing EBV+ from EBV- samples.

The results of the ROC analysis identified 320 genes with AUC above 0.75 (**Supplementary Table 4**). The genes *LGALS17A* (AUC = 1), *GBP5* (AUC = 0.99), *C1QC* (AUC = 0.99), *C1QA* (AUC = 0.98), *C1QB* (AUC = 0.97), *CMKLR1* (AUC = 0.97), *CXCR2P1* (AUC = 0.97), *GM2A* (AUC = 0.97), *CXCL11* (AUC = 0.97), *CD300LF* (AUC = 0.96), *IL32* (AUC = 0.96) (**Figure 3**) discriminated EBV+ patients with high sensitivity and specificity, suggesting they are potential biomarkers for identifying patients with EBV in gastric cancer.

Among the listed genes, *LGALS17A* showed the highest AUC value (1), which is related to the very low expression of this gene in EBV- compared to EBV+ patients (p<0.0001), suggesting that this gene has an important role in the pathogenesis of EBV, which needs to be clarified. Importantly, there is no literature available regarding the function of this gene that substantiates its involvement with EBV and/or cancer.

Genes *C1QA*, *C1QB*, *C1QC*, *GBP5 CMKLR1, CXCL11* and *CXCR2P1* indicates that EBV may be activating inflammatory and immunomodulatory pathways in these tumors. Similarly, the elevated expression of *IL32*, an inflammatory cytokine, suggests that EBV could be driving a pro-tumoral inflammatory response.

### 3.3 KEGG enrichment pathways of DEGs

**Figure 4:**
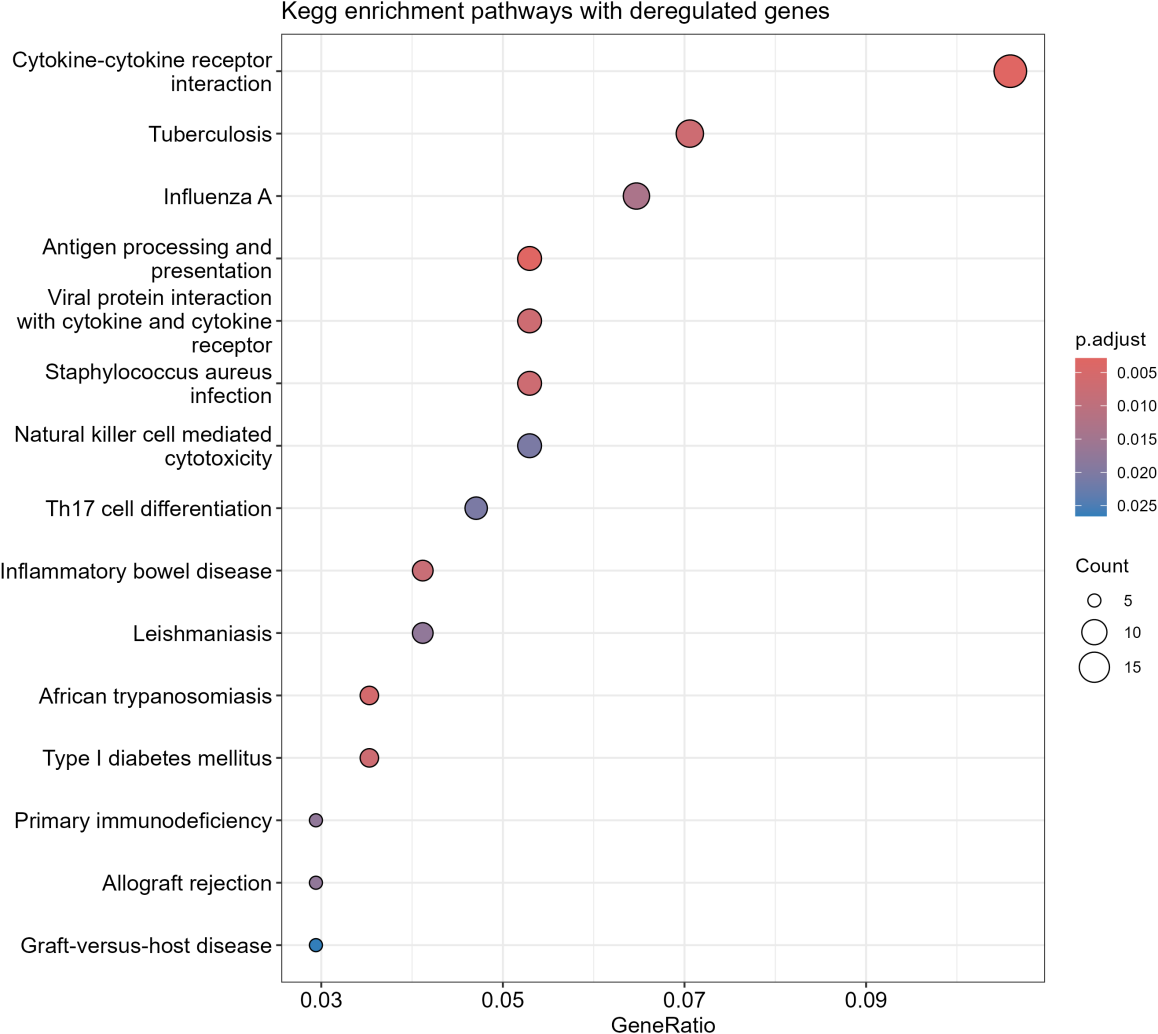
Kegg analysis of differentially expressed genes in EBV+ GC.

KEGG analysis revealed that many of the differentially expressed genes selected by the ROC curve are involved in immune-related processes, such as cytokine interaction, antigen presentation, Th17 response, and other pathways associated with opportunistic infections (**Figure 4**).

### 3.4 Differential expression analysis of bacterial genera

**Figure 5:**
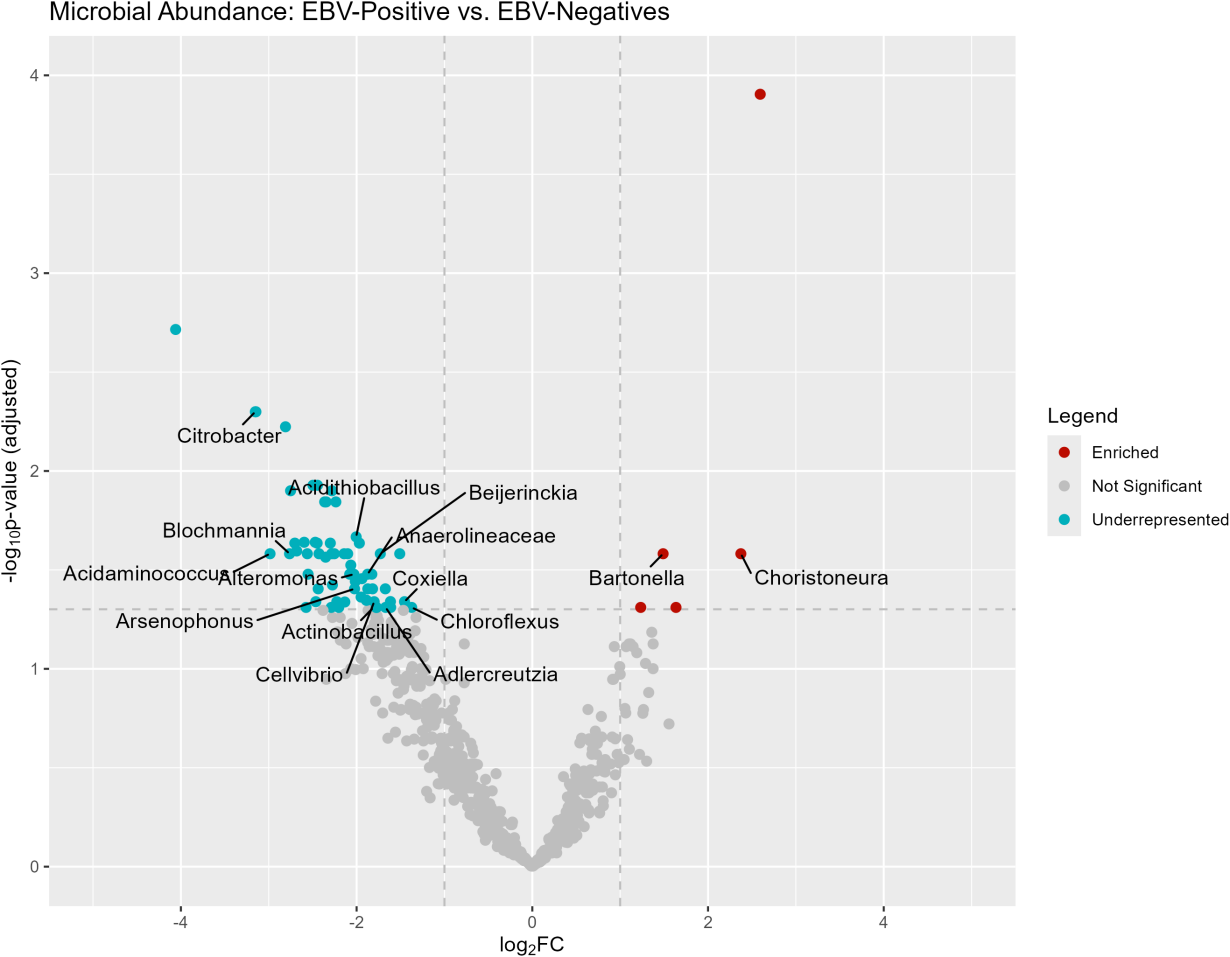
Differentially abundant genera in EBV+ and EBV- GC samples.

When examining the bacterial genera present in the samples, **Figure 5** displays differentially abundant bacteria in EBV+ and EBV- GC. The genera *Choristoneura* and *Bartonella* were more abundant in EBV+ GC, while differentially abundant bacteria in EBV- GC included *Citrobacter*, *Acidithiobacillus, Biochmannia, Beijerinckia*, and *Acidaminococcus*.

**Figure 6:**
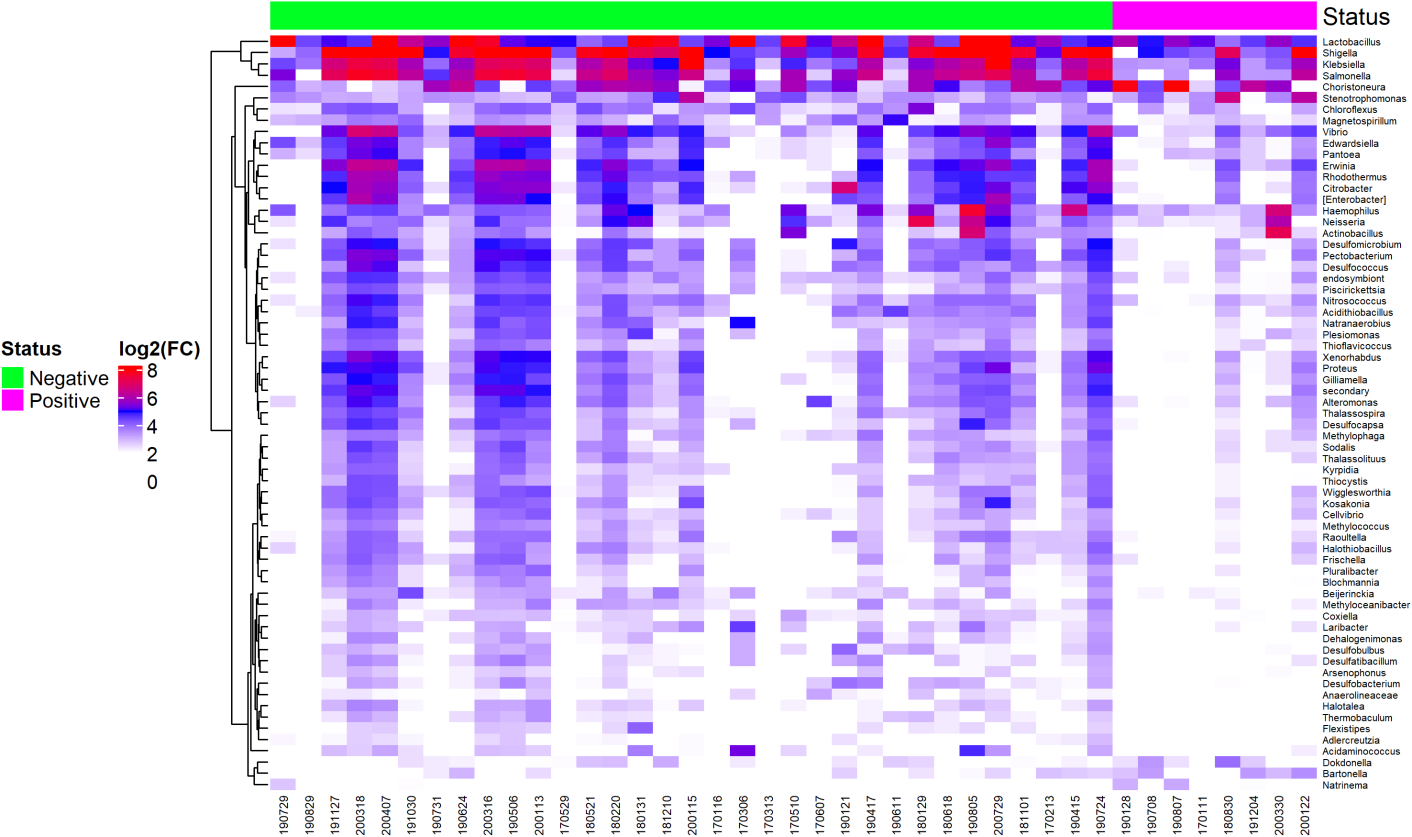
Heatmap of differentially abundant bacterial genera between EBV-positive and EBV-negative statuses.

Bacterial abundance in EBV-positive and EBV-negative GC samples is represented in a heatmap, showing that EBV+ samples had low abundance for most genera, while a small subset (*Shigella*, *Klebsiella*, and *Salmonella*) remained higher than other bacterial clusters (**Figure 6**). This same group also maintained high abundance in EBV-negative samples. See **Supplementary Table 2** for details.

**Figure 7:**
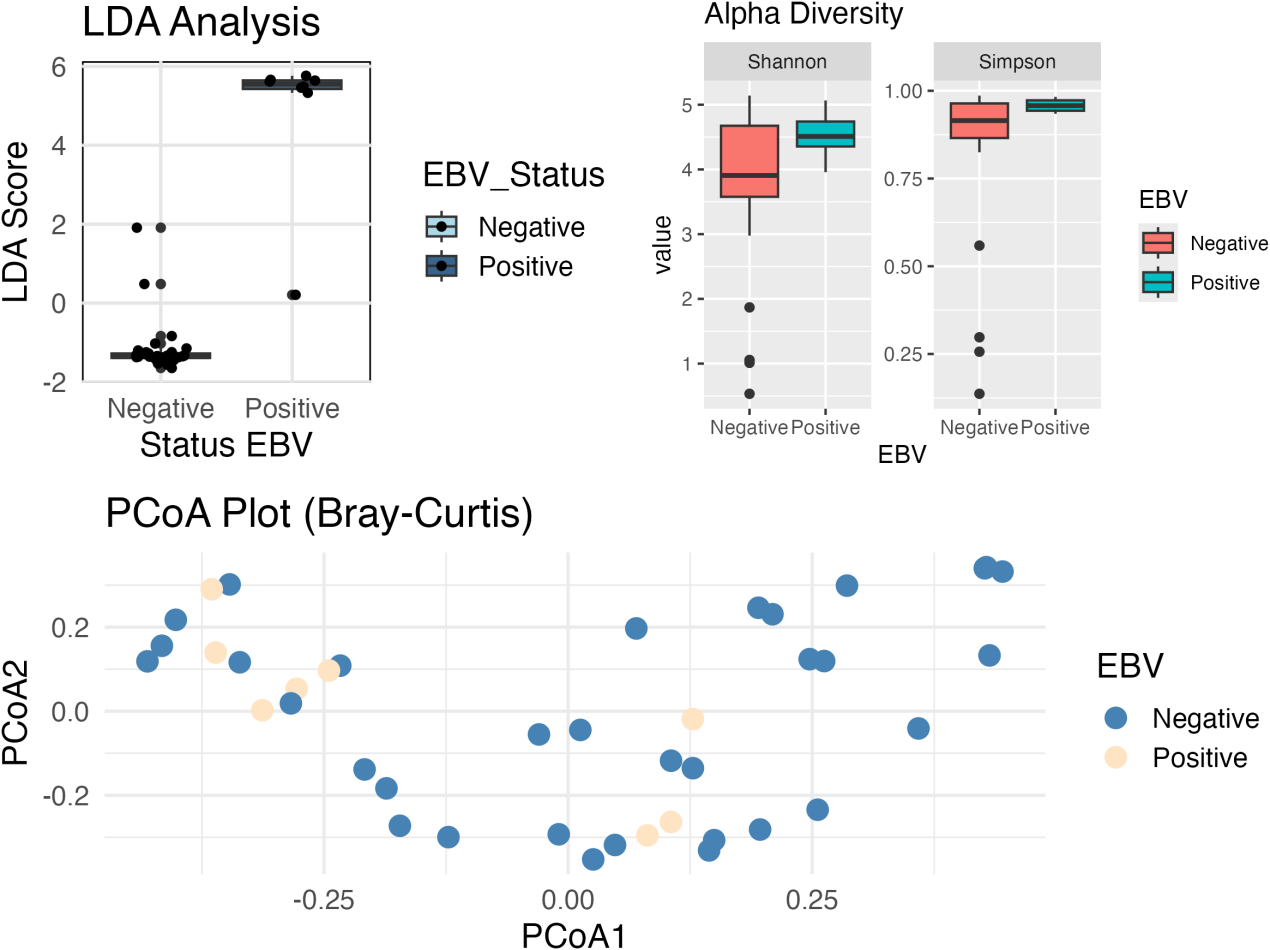
Boxplot comparing Linear Discriminant Analysis (LDA) scores between EBV-negative and EBV-positive samples. EBV-positive samples have higher LDA scores compared to the EBV-negative samples (p. value < 0.001), suggesting a clear separation between the two conditions. Alpha diversity analysis shows significant differences in the diversity and uniformity of bacterial communities between the groups, based on the Shannon (p-value = 0.038) and Simpson (p-value = 0.024) indexes. The Shannon index indicates that EBV+ patients have a more balanced distribution between species, reflecting less dominance of some species, while the Simpson analysis suggests lower bacterial diversity in this group. Beta diversity analysis demonstrate that the overall composition of the bacterial community (PCoA plot; PERMANOVA, p-value = 0.141) did not exhibit statistically significant separation between the groups, suggesting that both share a substantial overlap in taxonomic composition.

Additionally, we have conducted a Linear Discriminant Analysis (LDA) to assess the separation between the EBV-positive and EBV-negative groups based on bacterial abundances. The resulting LDA scores are visualized in the **Figure 7** (boxplot). As shown, the EBV-positive group exhibits significantly higher LDA scores compared to the EBV-negative group (Wilcoxon; p-value = 0.00001). This clear separation indicates that the bacterial abundance profiles differ between the two groups.

Alpha Diversity analysis (Shannon: p-value = 0.038, Simpson: p-value = 0.024) demonstrated that EBV+ and EBV- patients have significant differences in terms of alpha diversity (diversity within each group).

Results indicate that bacterial communities in EBV+ samples differ in uniformity (Shannon) from EBV-, as it has a more balanced distribution between species, that is, less dominance of one or a few species. Additionally, Simpson’s analysis demonstrates that the EBV+ group has a lower bacterial diversity.

No statistically significant separation was found between groups in the overall composition of the bacterial community (PERMANOVA, p-value = 0.141). This could mean that, although there are significant differences in bacterial uniformity and diversity within groups (alpha diversity), the overall composition does not show a clear separation between groups, as they share many taxa.

This analysis strengthens our findings and highlights the distinct bacterial composition associated with EBV status in GC samples.

### 3.5 Correlation between DE genes and bacterial genera

**Figure 8:**
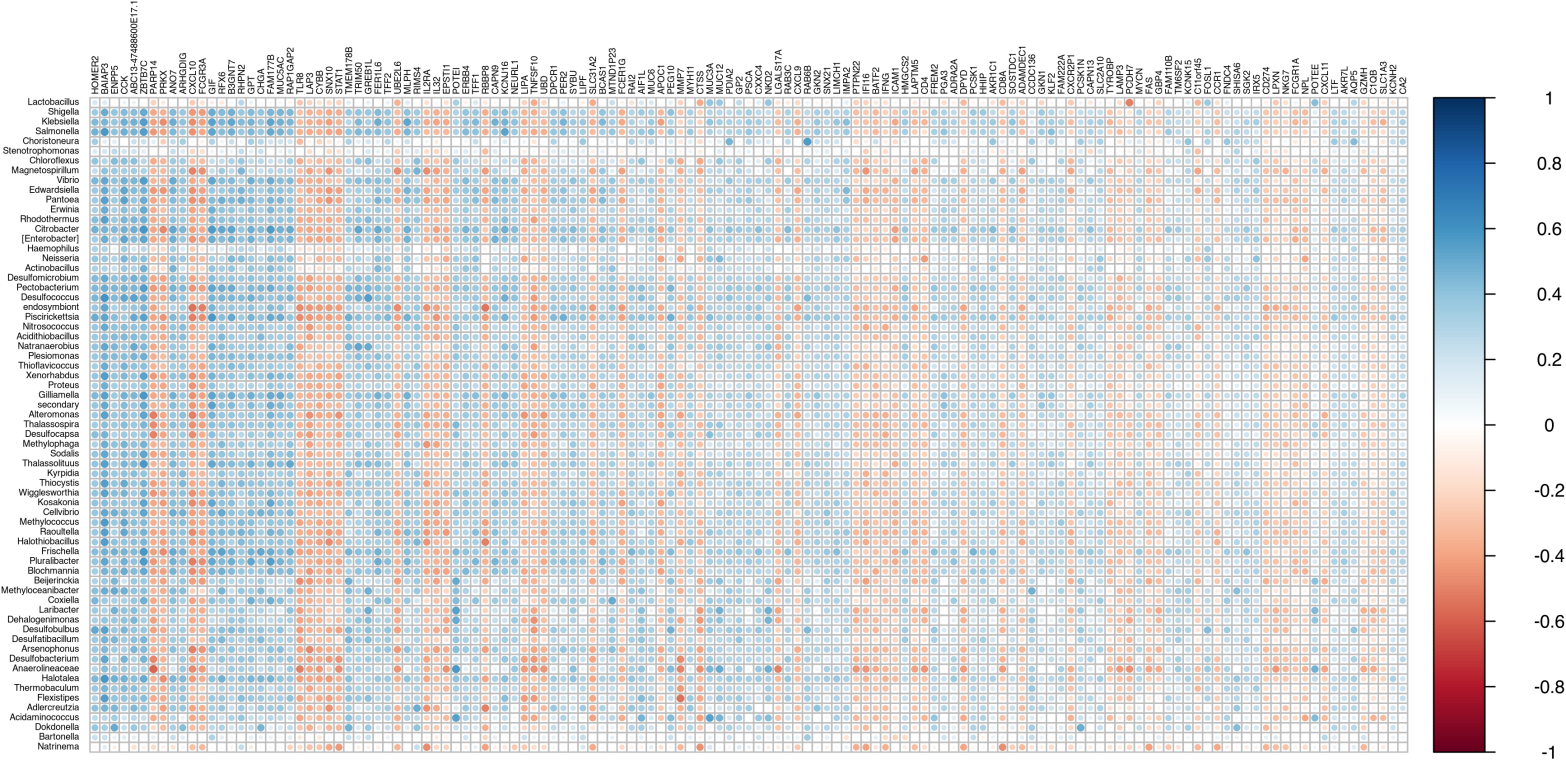
Correlation plot between differentially abundant bacteria and differentially expressed genes. (ρ > |0.3|) with at least 10% of DE bacteria.

One hundred and thirty-five DE genes were found correlated (ρ > |0.3|) with at least 10% of DE bacteria (**Figure 8**, **Supplementary Table 5**). The results suggest that alteration in bacterial abundance may be influencing or being influenced by the expression of these genes.

**Figure 9:**
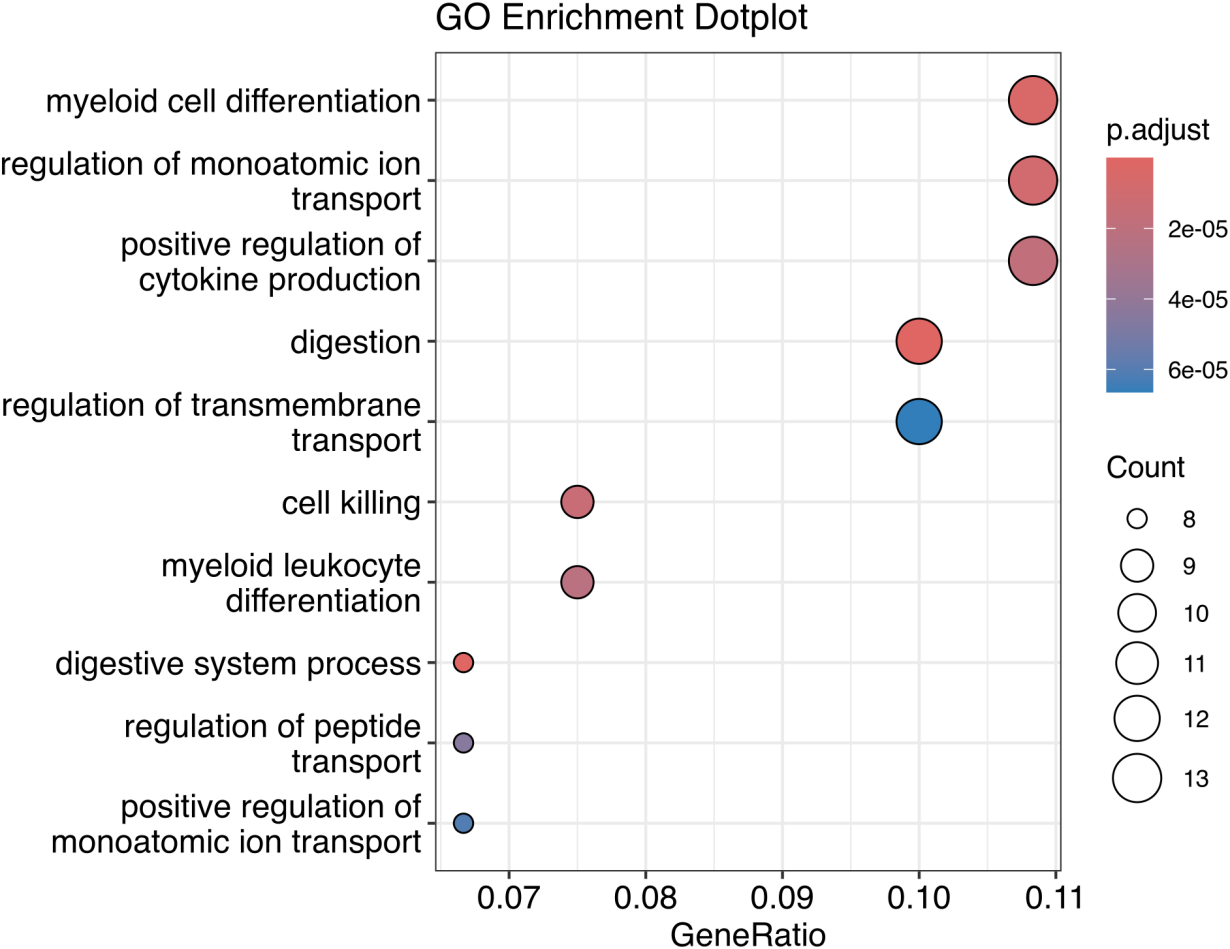
Gene Ontology of DE genes correlated to DE bacteria genera.

A gene ontology analysis was performed using the 135 genes to identify the biological processes involved (**Figure 9**). The main biological processes described indicate the participation of genes in cell differentiation, regulation of cytokine production, digestion and cell death, suggesting that these pathways can be modulated by the presence of EBV and the bacterial microbiota in the tumor microenvironment.

## 4 Discussion

The data analysis generated a heatmap indicating a cluster pattern for genes related to immune response in EBV-positive GC patients, including *IRF1*, *C1QB*, *TNFSF10* and *C1QC*. The relationship between EBV infection and immune system hyperactivation in the tumor environment is well-established, showing upregulation of various genes positively correlated with immune response and downregulation of genes negatively correlated with immune response (27).

In an immunocompetent host, both innate and adaptive immune responses are triggered by EBV infection, suppressing viral replication. EBV infection increased the proportions of T cells, cytotoxic lymphocytes, CD8+ T cells, NK cells, monocyte lineage cells, and myeloid dendritic cells. In EBV-negative tissues, neutrophils and fibroblasts were present in higher proportions (28, 29).

Importantly, Zhou et al. (29) identified *IRF1* (interferon regulatory factor 1) as a key immune-related gene in EBV+ GC, showing increased expression in samples. *IRF1* acts as a tumor suppressor and encodes a transcription factor that plays a key role in the body’s defense against infections, cell proliferation, and immune responses. It’s role in various human tumor types has important implications for cancer progression (30).

This gene regulates the expression of multiple genes central to both innate and adaptive immunity, indicating that *IRF1* may link the two systems. In addition to its functions in differentiated immune cells, *IRF1* has been shown to play roles in the development of various immune cells (dendritic cells, NK cells, CD8+ T cells) that, when activated, counteract the carcinogenic process (31).

The discussed observations demonstrate that tumor surveillance by the immune system is compromised by *IRF1* loss. Therefore, *IRF1* may function as a “systemic guardian”, protecting the host against exogenous mutagens that can lead to carcinogenesis (30). These findings support literature evidence and clinical data, including TCGA analyses, which indicate a strong correlation between EBV positivity and a more favorable prognosis (32, 33).

Moreover, the volcano plot illustrates differential expression of several genes in EBV-positive patients, notably *IL32* and *C1QA*, both associated with immune activation and also observed as upregulated in the heatmap.

EBV has the potential to immortalize B cells and infect epithelial cells. During immortalization, several EBV products induce cytokines or chemokines necessary for proliferation in infected cells. *IL-32* is upregulated after EBV infection, with EBV’s latent membrane protein 1 (*LMP1*) responsible for inducing its expression (34).

Interleukin *(IL)-32* is a recently discovered cytokine with potent pro-inflammatory activity. It is expressed in natural killer cells, monocytes, T lymphocytes, peripheral blood mononuclear cells, epithelial and endothelial cells, and fibroblasts, with various isoforms exhibiting different biological activities. *IL-32* is involved in establishing an inflammatory loop that, in turn, induces the synthesis of *IL-1β, TNFα, IL-6, IL-8*, and macrophage inflammatory protein *(MIP)-2* (35). Consistently, *IL-32* involvement has been documented in infectious diseases, chronic inflammatory conditions, including gastritis, inflammatory bowel disease, and cancer (36–38).

*IL-32’s* biological activity depends on cell type and context, with isoform-specific variations, allowing it to either promote or inhibit cancer development. Interestingly, *IL-32* and the mechanisms regulating its endogenous isoforms are considered potential targets for anti-neoplastic strategy (35, 36, 38, 39).

The complement system is a critical part of the immune system, protecting the body from invading bacteria and deleterious immune complexes via its enzymatic cascade, receptors, and proteins. Of complement constituents, *C1q* is a key molecule activating the classical pathway, leading to opsonization and phagocytosis. *C1q* is encoded by a cluster of three genes (*C1QA*, *C1QB*, and *C1QC*), with *C1QA* encoding the A-chain polypeptide of the serum complement subcomponent *C1q*, which associates with *C1r* and *C1s* to form the first component of the serum complement system (40).

Corroborating our finds, Deng et al. (41) identified *C1QA* as a differentially expressed gene when comparing EBV-positive GC patients and EBV-negative GC patients. An integrated bioinformatics analysis revealed that EBV-positive GC expressed more immune-related genes, including common immune checkpoints and human leukocyte antigen (HLA) genes, than EBV-negative GC. Immune scores were higher in EBV+ GC, indicating greater immune cell infiltration and identifying *C1QA* as an EBV+ GC biomarker.

In line with heatmap results, certain genes, including *FAM3B*, showed low expression in EBV-positive samples.

Liang et al. (42) studied the EBV+ GC mechanism, identifying 216 downregulated genes by EBV-induced hypermethylation, significantly increasing methylation of *ACSS1*, *FAM3B*, *IHH*, and *TRABD* in EBV-positive tumors.

DNA methylation is arguably the most significant mechanism in EBV-positive GC. Studies that promoters of 886 genes involved in cancer-related pathways were abnormally hypermethylated in EBV-positive GC (AGS) cells, including *FAM3B* (43–46).

Maciel-Silva et al. (47) reported that *FAM3B* could inhibit TNF-α-mediated programmed cell death by upregulating anti-apoptotic Bcl-2 family members and reducing caspase-3 proteolytic activity. These results suggest that *FAM3B* influences prostate tumorigenesis by modulating the expression of the cell survival genes Bcl-2 and Bcl-XL.

In their study, increased expression of *FAM3B* was found in AGS/CDDP cells, where its overexpression induced cisplatin resistance. Conversely, *FAM3B* knockdown increased cisplatin sensitivity in AGS/CDDP cells. Furthermore, *FAM3B* promoted epithelial-mesenchymal transition (EMT) in gastric cancer cells by upregulating snail protein. Snail inhibition reversed the EMT and cisplatin resistance induced by *FAM3B* (47).

*FAM3B* plays a pivotal role in cancer initiation and progression. When upregulated, it can promote invasion and metastasis in human colon cancer, esophageal squamous cell carcinoma, and prostate cancer cells and is associated with poor patient prognosis (47). Similarly, elevated *FAM3C* is strongly linked to poor prognosis in various cancers, including liver, colorectal, gastric, breast, esophageal squamous cell carcinoma, and oral squamous cell carcinoma (48).

These findings reinforce the oncogenic role of *FAM3B*. However, its downregulation in EBV-positive samples aligns with clinical and scientific evidence suggesting that EBV infection in gastric cancer is associated with a favorable prognosis.

ROC analysis identified several genes with high specificity and sensitivity for discriminating EBV+ samples in GC. Among these, the *LGALS17A* gene stood out with the highest AUC value, suggesting that it is a reliable biomarker for identifying patients with these conditions. However, despite its potential significance, *LGALS17A* is a pseudogene that has not yet been studied in the literature.

The *GBP5*, *C1QC*, *C1QA*, *C1QB*, *CMKLR1*, *CXCR2P1*, *GM2A*, *CXCL11*, and *IL32* genes play crucial roles in various physiological and pathological processes, including immune response, inflammation, and cancer (34, 35, 40, 41, 49–51). These genes also presented high AUC values (above 0.96), indicating that, together, they form a set of potential biomarkers, providing a more comprehensive understanding of the pathological characteristics of EBV-associated gastric cancer, that could contribute to enhanced diagnosis, prognosis, and the development of personalized therapies.

The *CXCL11* gene presents robust data in the literature, and recent studies have found that this gene is involved in the activation of multiple oncogenic signaling pathways and is closely related to tumorigenesis, progression, chemotherapy tolerance, immunotherapy efficacy, and poor prognosis (51–53). Notably, Zhang et al. (54) identified the *CXCL11* gene as a key factor in the upregulation of PD-L1 expression in gastric cancer (GC) cells, mediated through the activation of the STAT and PI3K–Akt signaling pathways.

Providing a deeper understanding of the gene expression landscape associated with the immune system, the functional enrichment analysis of differentially expressed genes highlighted cytokine-cytokine receptor interaction, antigen processing and presentation, as well as the Th17 immune response. These findings reinforce the role of the tumor microenvironment, shaped by inflammation and immunomodulation, in the pathogenesis of EBV-associated GC.

The cytokine-cytokine receptor interaction was the most enriched process, suggesting that cytokine-mediated signaling plays a central role in EBV-GC. Studies have demonstrated that EBV can induce the secretion of pro-inflammatory cytokines, such as IL-6 and TNF-α, which promote cell survival, angiogenesis, and tumor invasiveness. This pathway also contributes to local immunosuppression, facilitating tumor immune evasion (55).

The enrichment of antigen processing and presentation reflects EBV’s ability to subvert immune mechanisms. This virus can alter the expression of major histocompatibility complex (MHC) molecules, suppressing antigen presentation and, consequently, the activation of cytotoxic T cells (56). This immune evasion is crucial for viral persistence and tumor progression.

Furthermore, the activation of NK cell-mediated cytotoxicity suggests an initial innate immune response against EBV-infected cells. However, the virus has developed strategies to escape this surveillance, such as the expression of viral proteins that inhibit NK cell function (57).

Th17 differentiation has also emerged as an important aspect of the pathogenesis of EBV-associated GC, as well as other cancers like non-Hodgkin lymphoma (58). Th17 cells are known to mediate chronic inflammation, which is closely associated with the development and progression of gastric tumors (59). Studies indicate that EBV can induce an inflammatory phenotype that favors the expansion of Th17 cells, exacerbating the inflammatory tumor microenvironment (41).

Regarding the study of bacterial diversity present in patients with and without EBV, it was observed that the genera *Choristoneura* and *Bartonella* were significantly more abundant in EBV + patients.

These genera have not been previously associated with carcinogenesis. However, alterations in the microbiota are known to significantly impact gastric carcinogenesis. According to the literature, the normal gastric microbiota can harbor 128 phyla, though it is primarily dominated by five phyla: Proteobacteria, Firmicutes, Actinobacteria, Bacteroidetes, and Fusobacteria. *Helicobacter pylori* is the dominant bacterium in normal gastric mucosa and non-atrophic gastritis. Microbiome diversity tends to decrease when *H. pylori* predominates and increases as *H. pylori* diminishes, as seen in premalignant lesions and possibly in GC (60).

Generally, during gastric epithelial progression from normal to GC, *H. pylori* abundance decreases, while oral and intestinal microbiota frequencies and microbial diversity increase. At the phylum level, Firmicutes are significantly higher in GC patients (61). At the genus level, certain bacteria are enriched in GC patients, while others decrease, such as *Bacteroides*, *Verrucomicrobia*, *Deferribacteres*, and the *Lachnospiraceae*. Conversely, *Clostridium*, *Fusobacterium*, *Lactobacillus*, *Citrobacter*, *Achromobacter*, *Rhodococcus*, *Gemella*, *Pseudomonas*, and *Acidovorax* are elevated in GC patients.

Dysbiosis leads to microecological changes and activates inflammatory factors in the gastrointestinal mucosa, such as oxidative stress activation, nitric oxide (NO) release, and the production and secretion of pro-inflammatory cytokines and cyclooxygenase-2 (COX-2) (62).

Microbial data from this study reveal a significant decrease in bacterial abundance and diversity in EBV-positive GC samples, except for *Shigella*, *Salmonella*, and *Klebsiella*. These findings suggest a potential interaction between EBV infection and the gastric microbiota, contributing to the dysbiotic state observed in gastric cancer. Reduced bacterial diversity and abundance in EBV-positive samples may reflect the virus’s role in altering the gastric microenvironment, favoring immune evasion and tumor progression. EBV has been shown to modulate the immune system through cytokine signaling and inflammation, creating unfavorable conditions for commensal bacterial colonization (55). This dysbiosis may further exacerbate tumor-promoting processes by altering metabolic pathways and immune responses within the gastric niche.

Interestingly, the enrichment of *Shigella*, *Salmonella*, and *Klebsiella* in EBV-positive samples is noteworthy, as these genera are known for their pathogenic potential and association with inflammation. These bacteria have been implicated in gastrointestinal infections, and their persistence in EBV-positive GC samples may indicate a synergistic interaction between viral and bacterial factors, promoting a pro-inflammatory tumor microenvironment (63). For instance, *Shigella* and *Salmonella* can induce inflammatory responses via NF-κB activation (64) and other pathways, which may ultimately complement EBV-driven oncogenic processes.

These results align with emerging evidence linking viral infections to microbiota alterations in gastric cancer. EBV’s ability to suppress bacterial diversity while promoting specific pathogenic taxa underscores the complex interaction between microbial and viral factors in shaping the tumor microenvironment (65). Future studies should explore the mechanistic basis of these interactions, focusing on the role of microbial metabolites, immune modulation, and their contributions to gastric tumorigenesis (66).

In summary, gastric dysbiosis (encompassing bacteria, viruses, acid suppressants, antibiotics, and surgery) can lead to gastric immune dysfunction or result in a decrease in dominant bacteria and an increase in the number and virulence of pathogenic microorganisms, which, in turn, promotes GC development.

Finally, Spearman’s correlation demonstrated a strong correlation between DE bacterial genera and DEGs involved in the biological processes of cell differentiation, regulation of cytokine production, digestion and cell death.

Cellular differentiation and regulation of cytokine production show that these genes can influence the local immune response, which is relevant for understanding how gastric cancer can interact with chronic inflammation mediated by EBV and bacteria present in the tumor environment.

Modulation of digestion may suggest that microbial factors, together with altered gene expression, are implicated in altering gastric tissue homeostasis. Furthermore, cell death may indicate that these genetic and bacterial changes are involved in the survival of tumor cells, perhaps by affecting mechanisms of apoptosis or self-sufficiency in tumor growth.

Therefore, these findings suggest a complex biological process of interaction between the host (in this case, EBV+ gastric cancer) and the microbiota, possibly with a direct impact on cancer progression. These processes may also offer potential therapeutic targets, such as modulation of the microbiota or interventions in the regulation of the genes involved. Comparison with EBV- samples also helps to highlight those related to EBV-associated gastric cancer, providing additional insights into the specific influence of the virus and microbiota on the pathogenesis of gastric cancer.

## 5 Conclusion

From the transcriptomic analysis provided by this study, it was possible to investigate the transcriptional landscape of EBV-associated gastric cancer, where high expression of genes involved in immune processes (*GBP4, C1QA, ICAM1, CXCL11*) and pro-inflammatory processes (*IRF, IL32, TNF*) was observed in EBV-positive samples. This indicates that the presence of EBV may increase susceptibility to gastric cancer due to alterations in the immune response profile, initiating inflammatory processes as a contributing factor in the development of gastric carcinogenesis, particularly given the significant correlation between these genes and the EBV+ GC status.

Kegg analysis revealed the multifaceted role of EBV in reshaping the gastric tumor microenvironment, promoting pro-tumorigenic biological processes. Understanding these pathways provides valuable insights for the development of targeted therapies, such as cytokine inhibitors and immunotherapy strategies.

Additionally, microbiome analysis suggests that EBV infection in gastric cancer is associated with significant alterations in the gastric microbiota. While most bacterial taxa show reduced abundance in EBV-positive samples, specific pathogens like *Shigella*, *Salmonella*, and *Klebsiella* are enriched, potentially contributing to a pro-inflammatory and tumor-promoting microenvironment. This underscores the complex interaction between viral infection and microbial dynamics in gastric tumorigenesis.

Our findings indicate that both viral and bacterial factors play interdependent roles in modulating the tumor microenvironment, affecting immune response and gastric homeostasis. These results open possibilities for the development of innovative therapeutic strategies, such as microbiome modulation or interventions targeting key genes involved in this complex biological process, aiming to improve the diagnosis and treatment of EBV-associated gastric cancer.

## Data Availability

All relevant data is contained within the article: The original contributions presented in the study are included in the article/**Supplementary Material**, further inquiries can be directed to the corresponding author.
